# Intraday adaptation to extreme temperatures in outdoor activity

**DOI:** 10.1038/s41598-022-26928-y

**Published:** 2023-01-10

**Authors:** Yichun Fan, Jianghao Wang, Nick Obradovich, Siqi Zheng

**Affiliations:** 1 Sustainable Urbanization Lab, Department of Urban Studies and Planning, and Center for Real Estate, Massachusetts Institute of Technology, Cambridge, USA; 2 State Key Laboratory of Resources and Environmental Information System, Institute of Geographic Sciences and Natural Resources Research, Chinese Academy of Sciences, Beijing, China; 3 Project Regeneration, Mill Valley, USA

**Keywords:** Climate-change adaptation, Climate-change impacts, Environmental economics, Environmental impact, Psychology and behaviour

## Abstract

Linkages between climate and human activity are often calibrated at daily or monthly resolutions, which lacks the granularity to observe intraday adaptation behaviors. Ignoring this adaptation margin could mischaracterize the health consequences of future climate change. Here, we construct an hourly outdoor leisure activity database using billions of cell phone location requests in 10,499 parks in 2017 all over China to investigate the within-day outdoor activity rhythm. We find that hourly temperatures above 30 °C and 35 °C depress outdoor leisure activities by 5% (95% confidence interval, CI 3–7%) and by 13% (95% CI 10–16%) respectively. This activity-depressing effect is larger than previous daily or monthly studies due to intraday activity substitution from noon and afternoon to morning and evening. Intraday adaptation is larger for locations and dates with time flexibility, for individuals more frequently exposed to heat, and for parks situated in urban areas. Such within-day adaptation substantially reduces heat exposure, yet it also delays the active time at night by about half an hour, with potential side effect on sleep quality. Combining empirical estimates with outputs from downscaled climate models, we show that unmitigated climate change will generate sizable activity-depressing and activity-delaying effects in summer when projected on an hourly resolution. Our findings call for more attention in leveraging real-time activity data to understand intraday adaptation behaviors and their associated health consequences in climate change research.

## Introduction

Weather and climate have profound impacts on human health^1–4^. Non-optimum temperature is associated with a wide range of chronic diseases^5,6^, explains substantial portions of mortality worldwide^7^, and creates notable mental distress^8–10^. Besides the direct physical and psychological stress, weather conditions also mediate the human behaviors critical for human health outcomes, including physical activity^11^, sleep^12,13^, civil conflicts^14–17^, and suicide^18^. Tackling climate change has thus been considered as the greatest global health opportunity for the twenty-first century^19^.

A critical aspect of assessing the health and prosperity threats posed by climate change is to acquire knowledge about adaptation behaviors^20–23^. Previous studies have shown that human adaptation substantially reduces heat-induced mortality^24–26^. Researchers have made great efforts to classify individual adaptation behaviors^27^, to map their actual implementation^28^, and to study the psychosocial antecedents of adaptation^29^. Within-day time adjustment can greatly reduce heat exposure and thus constitute an important channel of behavioral adaptation in response to the risk of high heat stress^30^. Though the potential linkage between extreme temperature and the intra-day adjustment in outdoor activity timing is hypothesized^26,31^, there remains limited quantitative evidence on whether and to what extent people use intraday activity substitution as a behavioral adaptation strategy.

On the one hand, how extreme temperature affects the timing of outdoor activity determines actual temperature exposure. Robust projections of future heat-related health risks require considering both the hazardous environmental conditions and the cascade of health-relevant physiological and behavioral reactions^32^. Neglecting this intraday adaptation margin as a natural channel of human thermoregulation will overestimate population heat exposure risk and mischaracterize individual heat tolerance and preferences. On the other hand, activity timing is crucial for human rest-activity rhythm, a potentially omitted linkage between climate and health^33^. Delaying outdoor activity at night to avoid hot temperatures could contribute to nocturnal restlessness, a behavior pattern documented by biomedical research to be predictive of physical health, cognitive function, and subjective well-being^34^. As humans display rhythms in their physiology and behavior synchronized to environmental cycles of 24 h by being inactive at night to prepare for sleep^35,36^, disruption of normal rest-activity rhythm can also cause clinically relevant disorders, including neurodegeneration, diabetes, obesity, and cardiovascular disease^37–41^.

Existing literature predominantly relies on daily or monthly data and concludes that although extreme cold temperature substantially decreases outdoor activity, extremely hot temperatures have a small or statistically non-significant effect on outdoor activity^11,31,42–44^ (see Supplementary Table 1). With intra-day substitution as a behavioral adaptation channel, the level of daily outdoor activity hinges on the within-day temperature distribution rather than the daily average or maximum temperature adopted in previous studies.

To begin shedding light on the intraday adaptation margin, we compile a unique hourly activity dataset using 60 billion cell phone location inquiries per day all over China. For each hour, we query the activities within 10,499 parks, comprensively covering 19 categories of outdoor recreational sites. Parks are the most popular outdoor venues for people in China to conduct recreational and physical activities, providing synergic physical and mental health benefits^45^. Compared with self-reported surveys^46^ or entry records from a specific type of park^47–49^, our mobile phone position dataset also gives a more objective and representative documentation of hourly outdoor activity and are able to capture behaviors with large spatial and temporal variations in temperature to support causal identification. Coupling hourly data with econometrics, we provide the first empirical calibration of human within-day activity distribution patterns on days with different temperature distributions. We derive the activity-depressing and activity-delaying effects induced by extreme temperatures from our hourly estimates, which can support more accurate calibrations of public health consequences under future climate change.

## Methods

### Data

Our main mobile phone (MP) positioning data was acquired from Tencent’s location-based service (https://heat.qq.com), which contains the real-time geographic coordinates of more than 900 million users and more than 60 billion location requests per day all over China in 2017 (Supplementary Fig. 9). We have 10,499 parks all over China, including 19 categories. We calculate the number of cell phone location points within each park’s boundary on an hourly basis to formulate the panel data set of park visitation. We match the park-level hourly visitation index with hourly weather data from the closest station of the 2000 national meteorological monitoring stations. Weather variables comprehensively include temperature, precipitation, relative humidity, wind speed, wind direction, and air pressure. We further collect the cloud coverage data from MERRA-2, M2T1NXRAD project (https://disc.gsfc.nasa.gov/datasets/M2T1NXRAD_V5.12.4/summary). Meanwhile, we collect hourly air pollution data from 1500 pollution monitoring stations in China and apply the kriging spatial prediction method to interpolate the pollution level to each park. Finally, for heterogeneity explorations (results in Fig. 2), we collect city income from 2017 China City Statistical Yearbook and define whether a park is located in urban area through the Global Human Settlement Layer Urban Centres Database.

### Climate econometric model

We exploit the exogenous nature of hour-to-hour climatic variation along with rich sets of spatial and temporal fixed effects to identify the impact of extreme temperature on park visitation. Our preferred specification captures the instantaneous nonlinear effects of temperature by the following regression using ordinary least squares:

1 $$\log \left( {Y_{ict} + 1} \right) = f\left( {Temp_{ict} } \right) + X_{ict} \gamma + \delta_{i} + \eta_{h} + \theta_{dow} + \mu_{cm} + \varepsilon_{ct}$$

where *i* indexes park, *c* indexes the city the park falls in, *t* indexes time which is detailed into an hourly level. The outcome variable of interest $Y_{ict}$ is the visitation number for park *i* in city *c* at time *t*. Only hours between 6 AM and 10 PM is considered. We add 1 to the visitation index to avoid the zero visitation (zero visitation only accounts for 0.125% of the observations thus will not create bias to our estimation). Our relationship of interest is captured by $f\left( {Temp_{ict} } \right)$, which provides separate indicator variables for each 5 °C temperature bin, ranging from the lowest to highest temperature, enabling flexible estimation of a nonlinear relationship. Control variable $X_{ict}$ includes other weather variables (i.e., precipitation, wind speed, humidity, air pressure, and cloud coverage), air pollution (measured by the overall air quality index (AQI)), and a dummy variable indicating holidays. $\gamma$ indicates the regression coefficients of park visitation on the control variables. Taking advantage of the panel data structure, we include the park fixed effects ($\delta_{i}$), hour-of-the-date fixed effects ($\eta_{h}$), day-of-week fixed effects ($\theta_{dow}$), and city by month fixed effects ($\mu_{cm}$). This setting allows us to control for the unobservable time, spatial, and locally seasonal variation factors and exploit the exogenous daily fluctuations in temperature across the same park within the same hour overtime to identify the causal effect on visitation. The standard errors are clustered at the city level to non-parametrically adjust for arbitrary within-unit autocorrelation in the disturbance term $\varepsilon_{ct}$. The regression coefficients are adjusted by $e^{\beta } - 1$ to get the percentage change for interpretability.

### Activity time shift

The goal of calculating this “activity time shift”, is to understand on those days with extreme temperature, after what time point of the day *h* + Δ*h* would there be the same level of outdoor activity to that after time *h* on days with a comfortable temperature. The quantification requires two inputs: (1) daily park visitation activity quantity under different daily temperature scenarios; (2) within-day park visitation activity distribution by hour under different daily temperature scenarios (Supplementary Fig. 4). We can obtain the share of this activity taken place after hour *h* by dividing the sum of activity after time *h* with that of the whole day, each integrated the within-day activity distribution over the time span of interest. Multiplying the absolute activity quantity with the share of activity after time *h* gives the activity level after time *h* for days with different daily temperatures, denoted as *f* (*h* |T). We search for Δ*h* which can equalize *f* (*h* |T^0^ = 15–20 °C) with *f* (*h* + Δ*h* |T’).

Specifically, the calculation of activity time shift proceeds in three steps. First, we calculate the proportion of activity after a certain time *h*, *r*(*h*). Specifically, we transform the within-day activity distribution pattern (as shown in Fig. 4a) into the relative activity magnitude of each hour given temperature and then apply Eq. (2) to get the ratio of activity after time t with that for the whole day.

2 $$r\left( h \right) = {{\left( {\mathop \smallint \limits_{h}^{22} e^{\beta } dh} \right)} \mathord{\left/ {\vphantom {{\left( {\mathop \smallint \limits_{h}^{22} e^{\beta } dh} \right)} {\left( {\mathop \smallint \limits_{6}^{22} e^{\beta } dh} \right)}}} \right. \kern-0pt} {\left( {\mathop \smallint \limits_{6}^{22} e^{\beta } dh} \right)}}$$

**Figure 1:**
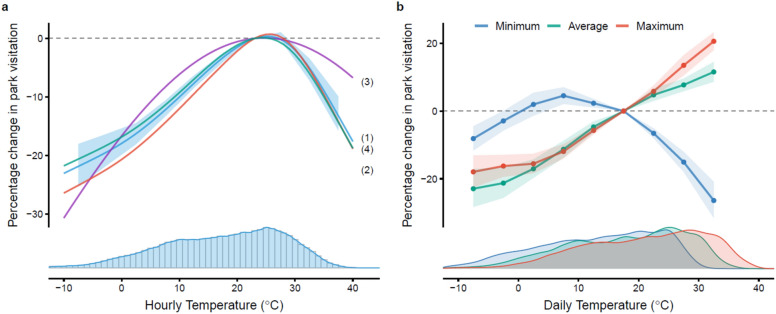
Temperature effects on park visitation quantity. (**a**) Effects of hourly temperature on park visitation with different model specifications. (1) Primary setting: City-month, day-of-week, hour-of-day fixed effect (FE) + binned regression with 5 °C intervals; (2) date FE in replacement of day-of-week FE; (3) quadratic polynomial; (4) binned regression with 3 °C intervals. (**b**) Effects of daily minimum, average, and maximum temperature (6 AM to 10 PM) on park visitation. Shaded areas are 95% confidence intervals. Histogram/Density plots display the distribution of temperatures for each sub-sample.

Second, we build a functional relationship between daily activity quantity and temperature, *q* (T). The mapping is obtained through the same econometric specification as our main results in Fig. 1, yet with daily instead of hourly observations as input. We then transform the effect of temperature on the quantity of daily activity (Supplementary Fig. 1) into the relative daily activity magnitude for each temperature with 15–20 °C as the baseline.

Third, we multiply the activity share *r*(*h*) with the daily activity quantity *q* (T) to obtain the activity quantity after time *h*. We set the accumulated activity quantity after 8 PM on days with average temperature 15–20 °C as the baseline, and perform the optimization process to obtain the time shift Δ*t*, for which the accumulated activity after 8 + Δ*h* PM equals that after 8 PM on days with temperature 15–20 °C.

3 $$r\left( {8 PM; 15 - 20^\circ C} \right) \times q\left( {15 - 20^\circ C} \right) = r(8 + \Delta h PM; > 30^\circ C) \times q( > 30^\circ C)$$

We choose 8 PM since the daily rise of melatonin secretion usually takes place around this time to prepare people for sleep^50^, and thus physical activity after 8 PM might negatively impact sleep.

### Future prediction of climate impacts

The climate prediction data are drawn from the Coupled Model Intercomparison Project 5 (CMIP5). We utilize the projections from 21 climate models under the RCP 8.5 emissions scenario as our main inputs for future climate change. We process data from each climate model separately to enable the calculation of bootstrap standard error. For each of the 21 models, the projected temperature changes in monthly temperatures are obtained for each 2.5 × 2.5-degree grid cell by differencing the monthly projected temperature in 2050–2059 (or 2090–2099) with that of 2017 (i.e., our baseline year). Then we convert from a 2.5 × 2.5-degree grid to a 25 × 25 km grid cell using the area-to-point downscaling method^51^ via the atakrig package^52^ to match historical weather data.

Monthly averages of projected temperature changes are temporally disaggregated into hourly realizations using historical weather variability in 2017, in accordance with the method adopted by previous research^53^. Specifically, for each month, we use the observed average temperature fluctuation in 2017 for each hour as the base and add the projected monthly temperature changes from the projection models:

4 $$T_{h,m,y} = T_{h,m,2017} + \Delta T_{m,y}$$

where $T_{h,m,y}$ is the average temperature for hour *h* of the day (e.g., 8 PM) in month *m* of year *y*, $T_{h,m,2017}$ is the average realized temperature for hour *h* of the day in month *m* of 2017, and $\Delta T_{m,y}$ is the projected daily average temperature change in month *m* of year *y* compared with 2017 according to the climate model. Here we need to assume temperature increase is the same for all hours within a day due to the lack of data for projected temperature change by each hour *h*.

We fit a natural cubic spline to our estimated dose–response function between binned temperature and activity quantity (Fig. 1) to obtain a continuous mapping of hourly temperature on park visitation (Supplementary Fig. 10a). We then combine the projected monthly average temperature for each hour into this fitted model to estimate the potential percentage change in park visitation due to global warming in each month of 2050–2059 (or 2090–2099). We also use a similar procedure yet with average temperature inputs by hour for summer (June to August) and winter (December to February) from the climate models to project the additional percentage point of activity change for each year from 2017 to 2099 to display the general trend of annual variation in seasonal activity rhythm. Similarly, we fit a cubic spline to our estimated relationship between temperature and time shift (Supplementary Fig. 10b) and input the projected temperature of 2050–2059 and 2090–2099 to calculate the projected time shift in intraday rest-activity rhythm.

The estimations of changes in both activity quantity and timing have multiple sources of uncertainty: the climate uncertainty, the econometric model uncertainty, and the uncertainty generated by the interaction of these factors. We adopt a similar strategy as previous climate econometric paper^54^ and use Monte Carlo sampling to account for both uncertainties. We characterize the climate realization uncertainty in 21 climate models and econometric uncertainty in 25 quantiles of each coefficient on the estimated response curve. We thus calculate 525 possible values for each impact dimension in each month in the future. The empirical distribution functions are used as impact distribution functions to obtain the 95% confidence interval.

## Results

### Temperature and activity quantity

Our research contrasts with past studies—we do document a large and significant activity-depressing effect on the high-temperature side of the distribution. We find that the contemporaneous impacts of hourly temperature on park visitation appear to have an inverted-U shape (Fig. 1a). The marginal effect of 1 °C deviation from the most comfortable temperature range (i.e., 20–25 °C) appears symmetric, yet with a long tail on the cold temperature side. Compared with 20–25 °C, hourly temperature below 0 °C decreases park visitation by 20% (95% confidence interval, CI 18–22%), and hourly temperature above 30 °C and 35 °C reduces park activity by 5% (95% CI 3–7%) and 13% (95% CI 10–16%) respectively. These estimates are much larger than the previous findings on the daily basis (see Supplementary Table 1) and robust under different specifications (Fig. 1a). We have summarized our estimated impacts of temperature, weather controls, and their interactions in Supplementary Table 3, 4. As expected, we find significantly negative impacts of precipitation, wind speed, and humidity on park visitation. And our preliminary tests suggest no clear evidence that other meteorological variables have interaction effects with extreme heat to jointly affect park activities.

To understand the mechanisms driving the differences between our analysis and previous studies, we examine the same climate econometric model with the same set of control variables yet use daily temperature, instead of hourly, as input. The results suggest that daytime (6 am to 10 pm) minimum temperature, rather than the average or maximum temperature (which are usually used in climate impact studies), determines the activity level individuals are willing to participate in response to temperature (Fig. 1b). This depicts the within-day substitution strategy people are taking to hedge against extreme temperatures. Even if the maximum or average temperature is high, as long as cooler periods of the day allow people to switch their activities within a day, we will not observe a significant decline in daily activity quantity.

**Figure 2:**
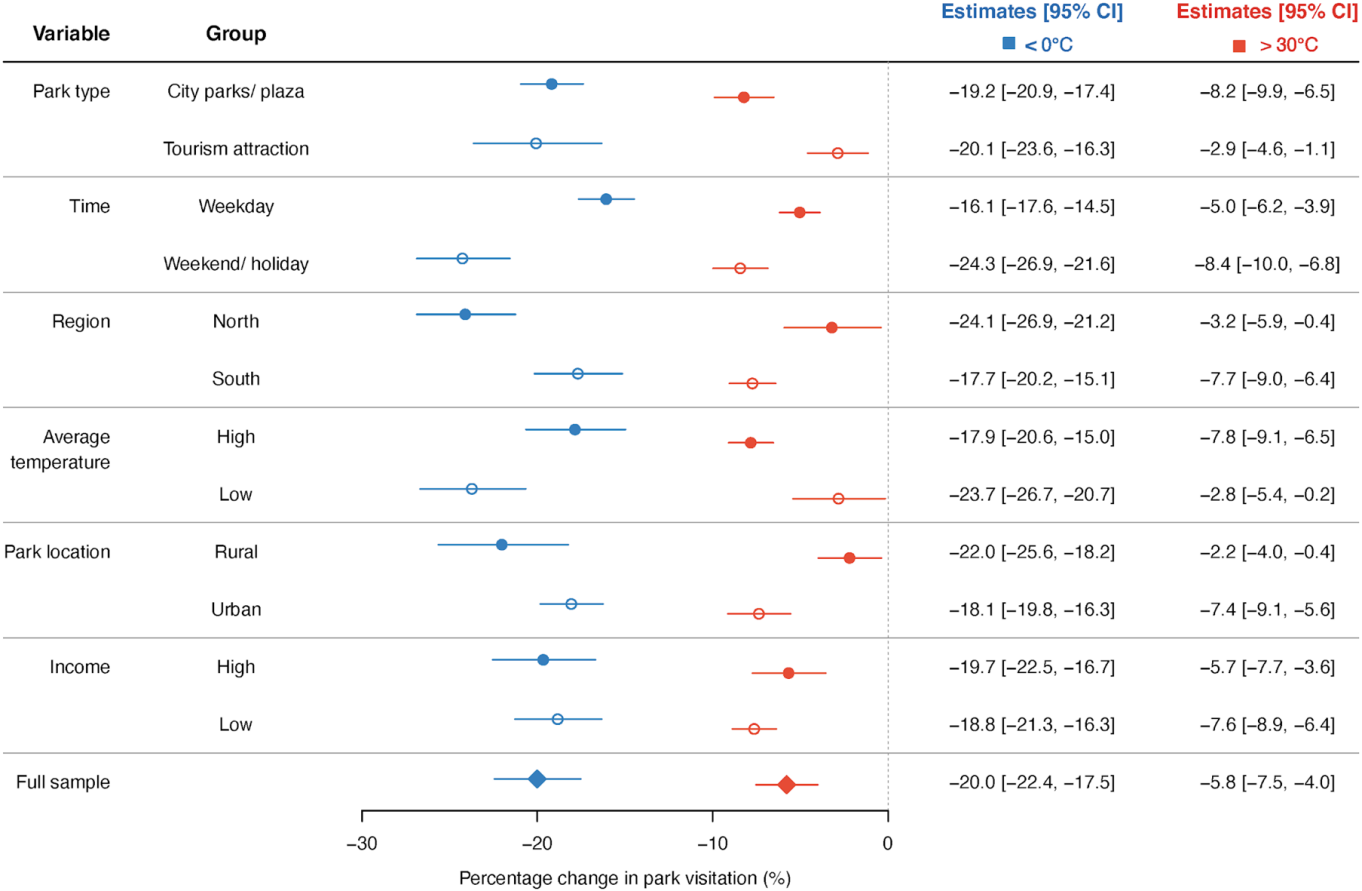
Heterogeneous effects of hourly temperature on park visitation quantity. Dots are point estimates when applying Eq. (1) yet merging the bins above 30 °C and below 0 °C. Error bars are 95% confidence intervals. Detailed heterogeneity analysis by temperature bins is summarized in Supplementary Fig. 2 and the numerical estimates are presented in Supplementary Table 5.

If the gap between our findings and the daily analysis of previous research is indeed driven by within-day temporal displacement of activity, we would expect to see higher sensitivity to excessive heat at parks where visitation time is more constrained and on days when the time flexibility is low. We stratify our sample by places and times to examine the heterogeneity in response elasticity (Fig. 2). Tourist attractions usually close at evening (i.e., the cooler times of a day). Thus, they lack the flexibility to switch activities to a cooler time of the day. We find clear evidence that visiting tourist attractions is less sensitive to extreme hot temperatures than normal city parks (Tourism attractions are usually gated parks with strict opening hours, while normal city parks are usually non-gated and open 24/7). Similarly, the time constraints on weekdays cause heat sensitivity to be significantly smaller than weekends.

We further test potential moderators on the temperature sensitivity of outdoor activity through a range of heterogeneity analyses (see Fig. 2). First, instead of finding evidence that individuals more frequently exposed to hot temperatures are less sensitive by making physical acclimation, we find higher sensitivity to hourly hot temperatures in southern China and in regions with higher temperatures. This result depicts a larger extent of temporal substitution in regions where the habits of avoiding the hottest period of day are more well-developed, suggesting the important role of behavioral adaptation. Second, parks situated in urban areas are exposed to hotter weather in temperature density distribution and, at the same time, more affected by extremely hot temperatures in visitation quantity. This provides suggestive evidence of the urban heat islands that cities modify the surface energy balance and intensify the adverse effects of hot temperatures^5,55^. In contrast, we find no evidence that income differences across cities mediate the temperature elasticity of park visitation.

### Intraday activity substitution as an adaptation strategy

**Figure 3:**
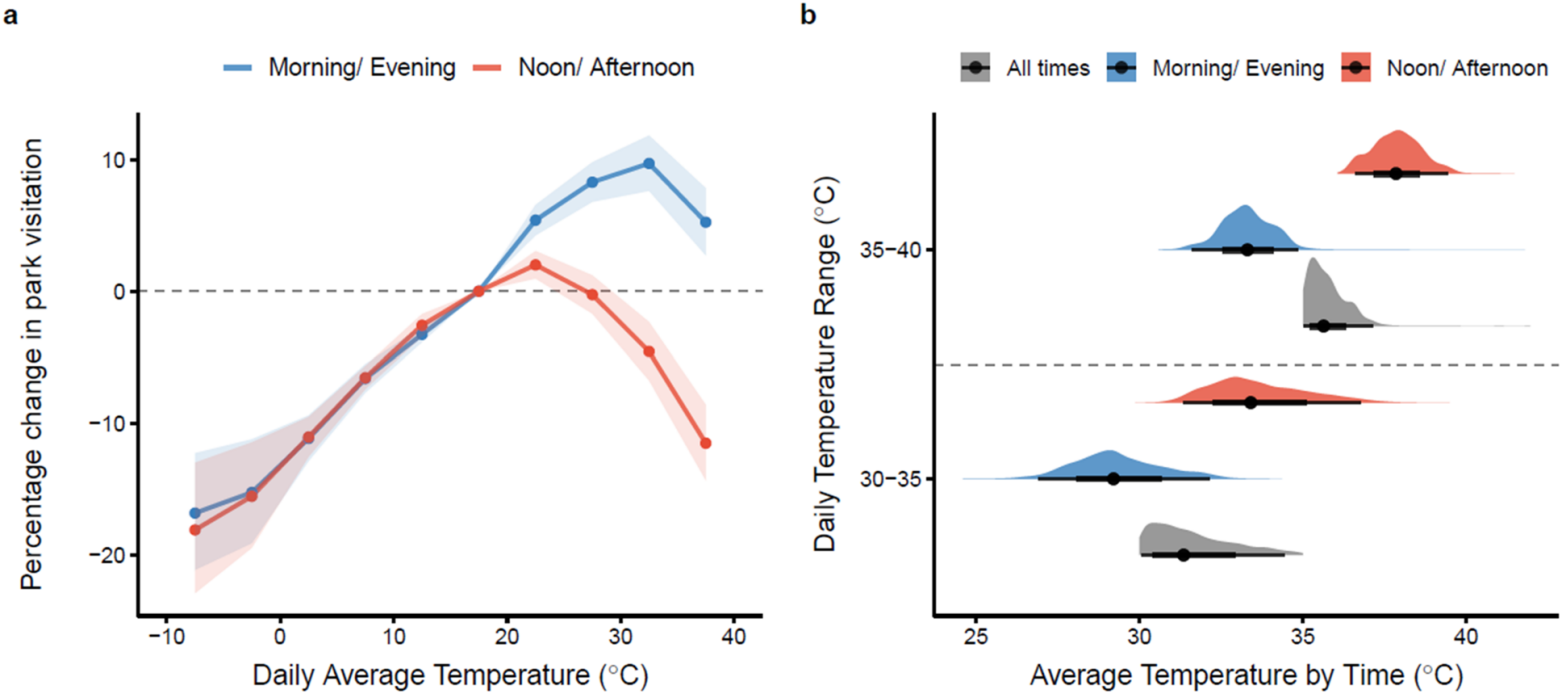
Activity and temperature distribution in hot and cool times of a day. (**a**) Effects of the daily average temperature on activities at different periods of the day. Shaded areas are 95% confidence intervals. Density plots display the distribution of temperatures for each sub-sample. **(b**) Average daytime temperature for different periods. Temperature distribution on days with the daily average temperature in 30–35 °C and 35–40 °C bins are presented, respectively. The point in each distribution is the median. The thicker and thinner lines show the interquartile range (i.e., 25–75%) and 95% confidence intervals. The colored shades display the density distribution.

To provide direct evidence on intraday temporal substitution as a short-term temperature adaptation strategy, we examine the impacts of temperature on activity quantity during different periods of the day. Similar to previous literature^42,56^, we decompose a day into hotter times [i.e., noon (10 AM–2 PM) and late afternoon (3 PM–6 PM)], cooler times [i.e., morning (6 AM–9 AM) and evening (7 PM–10 PM)]. We use the binned average daily temperature as the explanatory variable and separately run the regression in Eq. (1) for each two-time strata. We find a much larger intraday activity substitution as a compensatory strategy^57^ to hedge against hot temperature compared with cold temperature. When the weather is hot, people significantly reduce their activity level at noon and late afternoon while increasing those in the mornings and evenings (Fig. 3a). The results depict why daily data lacks the temporal granularity to document within-day adaptation mechanisms and might falsely conclude null or even positive impact of temperature on human activity.

Within-day temporal substitution of activity provides benefits of reducing extreme temperature exposures for flexible outdoor activities. When the average daytime temperature is within 30–35 °C and 35–40 °C bins, switching activities from the hotter times of the day to the cooler ones can reduce the average temperature exposure from 33.6 to 29.3 °C and from 37.9 to 33.3 °C respectively (Fig. 3b). Average daily ambient temperature is commonly used in public health and economic studies as the heat exposure measurement to building the dose–response functions between morbidity/mortality risks and temperature exposures^21^. Accounting for the within-day human activity pattern and its adaptation to ambient temperature environments can support building a more accurate heat exposure index to link with public health outcomes.

### Temperature-driven alteration in rest-activity rhythm

**Figure 4:**
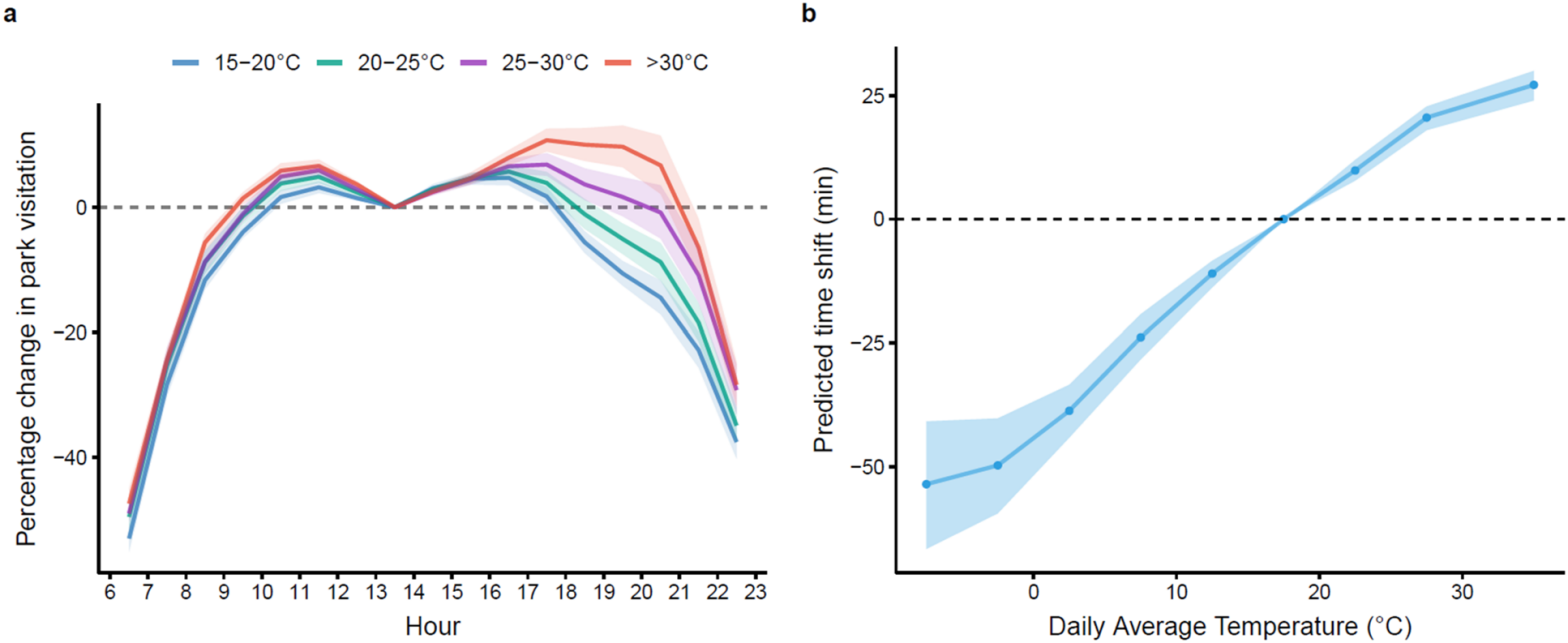
Quantification of temperature-driven time shift. (**a**) Within-day hourly activity distribution by daily average temperature range. Each line represents the within-day activity pattern for days lying in each daily average temperature range. Shaded areas are 95% confidence intervals. (**b**) Quantified time shift (Δ*t*) as a function of daily average temperature. Shaded areas are 95% confidence intervals calculated through Monte Carlo sampling of econometric parameter estimates of both the within-day activity distribution pattern (25 quantiles) and the daily activity quantity by temperature (25 quantiles)^54^.

To better understand how extreme temperature impacts the daily profile of activity patterns, we stratify the sample into groups according to the average daily temperature, set the mid-day (1 PM) as a reference for each temperature range, and add in hourly dummies in our main regression in replacement of temperature bins to explore the relative activity level at each hour compared to that of 1 PM. The results show a bimodal distribution of outdoor leisure activity. More importantly, it depicts that when faced with extremely hot temperatures, people significantly shift their activity towards nighttime (Fig. 4a). In contrast, the hourly distribution of activity remains in similar shapes on freezing days when compared with more temperate days, suggesting that intra-day adjustment in activity time is not a main adaptation channel to cold weather (Supplementary Fig. 3).

Sleep literature has shown that ending physical activity close to bedtime could negatively impact sleep quality^58,59^. We propose an approach to quantify the magnitude of temperature-driven nighttime activity delay to help illustrate its consequences. Suppose that conducting outdoor physical activity after time *t* would negatively affect sleep. The fundamental question we ask is: on days with extreme temperature, what time point *t* + Δ*t* would be equivalent to time point *t* on days with a comfortable temperature in sleep implications? We define equivalence as having the same level of outdoor activity after that time point. And we refer to Δ*t* as the shift in (sleep-relevant) activity time due to extreme temperature (see “Methods” for details).

We plot the activity time shift with 8 PM as the reference time in Fig. 4b. We set the reference as 8 PM since physical activity after this time could be considered as close to bed time. The monotonic dose–response function between activity time shift Δ*t* and daily average temperature suggests a continuous increase in late-night activity with hotter temperatures. When the average daytime temperature is above 30 °C, we observe the same level of outdoor activity of 27 min (95% CI 24–30 min) late compared with days with comfortable temperature. To what extent the delayed active time at night contributes to the negative effects of temperature on sleep requires more future studies.

### Projected activity rhythm under future climate change

Climate change poses a grave threat to human health. In this context, it is likely that future climate change will alter the adaptivity, frequency, and duration of human activity rhythms. To begin shedding light on the magnitude of impact, we project the potential impacts of future climate change on outdoor activity quantity and timing using the projected temperatures from the Coupled Model Intercomparison Project Phase 5 (CMIP5). We couple the predicted hourly temperature with our estimates of the relationship between temperature and park visitation to forecast the activity change (see Methods for details).

**Figure 5:**
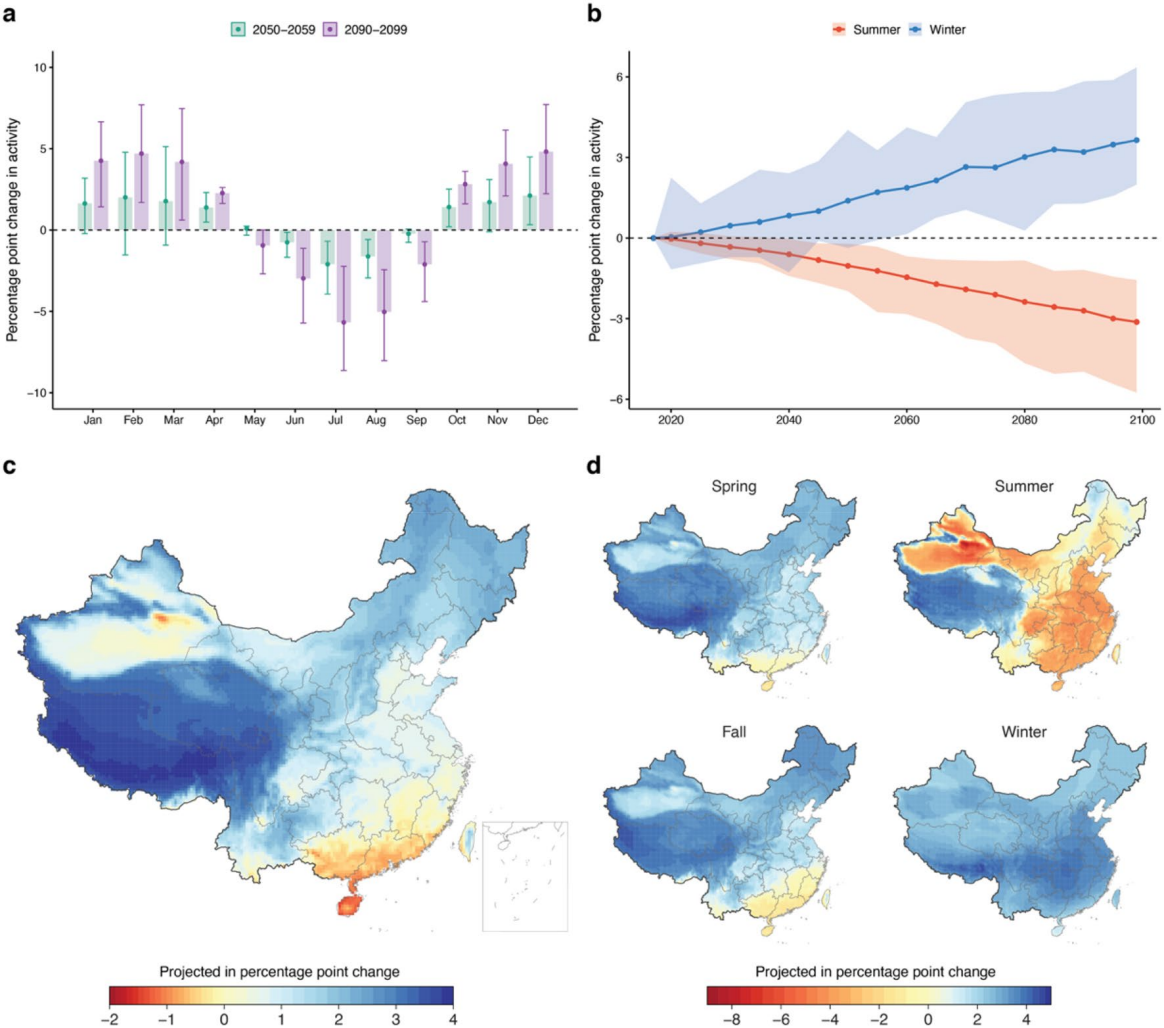
Projected change in park visitation quantity. (**a**) Additional percentage point change in park visitation quantity by month in the years 2050 and 2099 compared with 2017 due to climate change. (**b**) The percentage change in activity quantity for summer and winter respective for all years in the future. Geographic estimates of the projected change in park visitation quantity in all areas of China in 2090–2099 under the RCP8.5 scenario. Error bars and shaded areas represent 95% confidence intervals calculated through Monte Carlo sampling of econometric parameter estimates and climate models. (**c**) Geographic dispersion of the predicted additional yearly average effects of climate change-induced activity change in 2090–2099. **(d**) Prediction of additional climate change-induced activity change in 2090–2099 by seasons. Results for the RCP 4.5 scenario are presented in Supplementary Fig. 6. The figure was created using R (version 4.1.0 https://www.r-project.org/), and the maps do not have a copyright dispute.

Subtracting the activity changes due to temperature variation in 2050–2059 (or 2090–2099) from 2017 baseline, our hourly-based prediction suggests future climate change will intensify the seasonal variations in physical activity. We present the below projections under RCP 8.5, that reflect an "upper bound" of potential future changes in which emissions continue to rise throughout the twenty-first century. Results under a more moderate and currently realistic scenario, RCP 4.5, are presented in the Supplementary Material. We project an additional 5.7 percentage point reduction in July (the hottest month) and a 4.7 percentage point increase in February (the coldest month, due to warmer winter) on average in China by the end of this century (Fig. 5a). Such divergence across seasons continuously intensifies with time due to climate change (Fig. 5b). Although the yearly net effects is small due to the counteracting winter and summer impacts (Fig. 5c), global warming may cause an additional 4–10 percentage point loss in outdoor physical activity in the majority of areas of China in summer (Fig. 5d). The Qinghai-Tibet Plateau has a net increase in park visitation, yet the population density at that region is very low (see Supplementary Fig. 5). If we make a prediction based on daily average temperature using our data, we will falsely conclude that future climate change benefits physical activity even in the hottest season.

**Figure 6:**
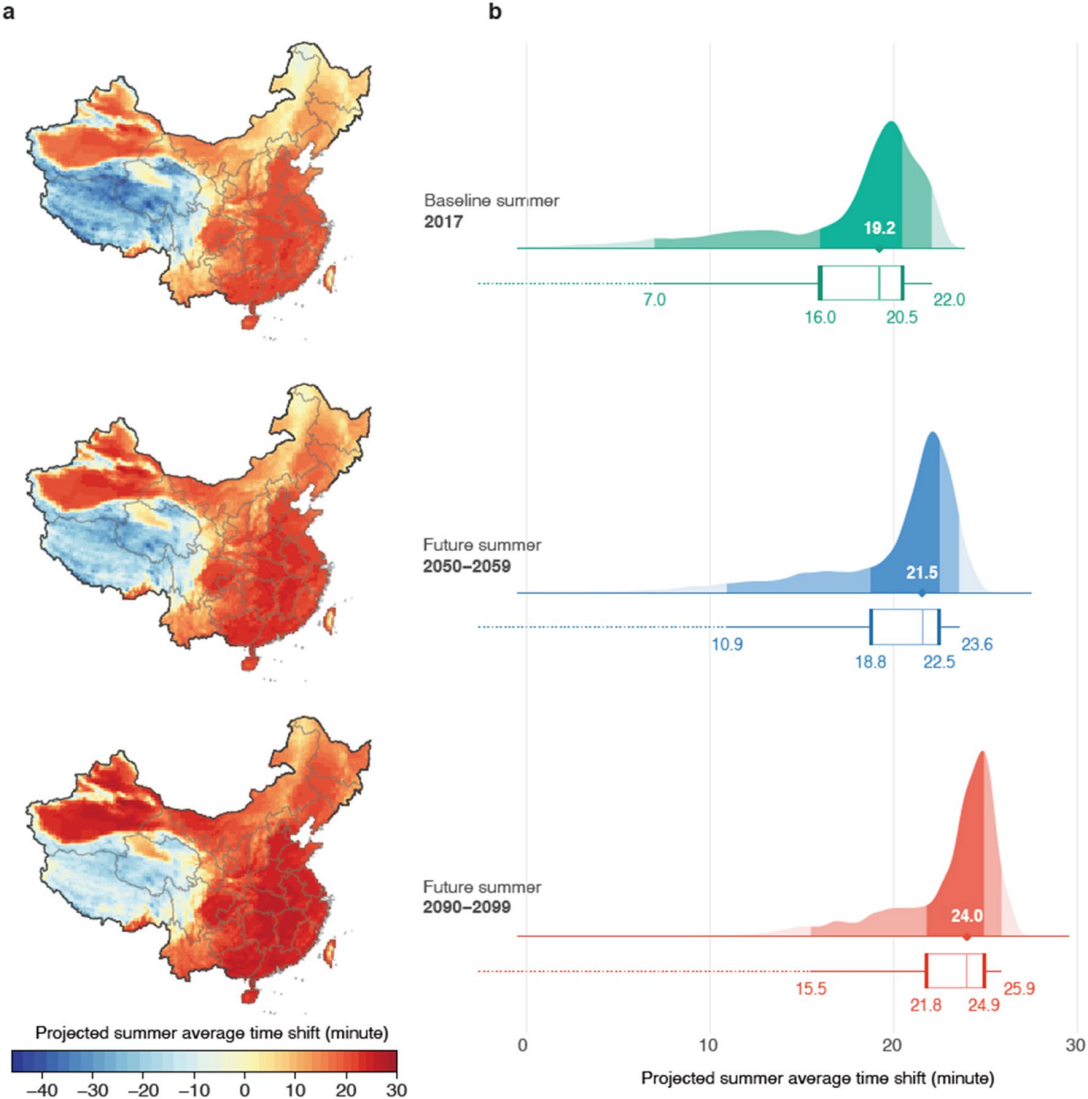
Projected time shift in intraday timing of activity in summer. (**a**) Geographic distribution of average yearly activity time shift due to temperature in 2017, at mid-century (2050–2059) and end of the century (2090–2099) under RCP8.5 scenario. (**b**) Size of populations subjecting to each yearly average time shift levels. Density plots how the distribution of time shifts across the population, and box plots show median, interquartile range (i.e., 25–75%) and 95% confidence intervals. The results displayed in each row are in 2017, at mid-century (2050–2059), and end of the century (2090–2099) under the RCP8.5 scenario. Results for yearly average of all seasons under RCP8.5 and RCP 4.5 are presented in Supplementary Fig. 7–8. The figure was created using R (version 4.1.0 https://www.r-project.org/), and the maps do not have a copyright dispute.

Besides the activity quantity, we project that outdoor activity rhythm will continue to shift towards nighttime in summer in response to global warming. More than 75% population in China will need to postpone outdoor activity time by on average 20 to 30 min towards nighttime to adapt to the hot climate (Fig. 6). Recent literature document that global climate change will likely increase daytime minimum temperature more than daytime average^60^. The differential temperature increase by time has not yet captured in our prediction model and is likely to further intensifies the activity-depressing and activity-delaying effects.

## Discussion

Extreme temperatures substantially depress observed human physical activities and alter within-day activity timing. The findings in previous literature conditions (as listed in Supplementary Table 1) concluding null or small impacts of hot temperature on human activity are unable to measure the intraday substitution humans commonly undertake to adapt to uncomfortable temperature conditions. Here we provide consistent evidence of how extreme hot temperature alters the within-day activity dynamic and develop a novel strategy to quantify the magnitude of activity delay.

Our results contrast with previous findings on climate change’s impacts on physical activity. While previous studies have suggested that future warming may benefit physical activity rates^11,42,43^, our findings indicate substantial negative effects of hot temperature. We find that individuals are not immune to hot temperatures, instead, they substitute their activity hours within day to avoid the hottest temperatures. As a result, if future climate change shifts up the whole distribution of temperature and drives up the daytime minimum temperature more than the maximum—as is expected^60,61^—it could deprive humans of the cool daily periods in hotter seasons that they currently shift their activities into. In contrast, we find limited adjustments in intraday activity time during the cold seasons. We conjecture that this difference is potentially driven by the lack of flexibility for leisure activities during the warm times of the day (i.e., around noon) compared with cool times of the day (i.e., evening and night). As improved health and fitness through physical activity can reduce heat stress risk^62^, more accurate projection of activity depressing effects due to intraday adaptation also has important health implications. Furthermore, beyond leisure time outdoor activities, our approach can be used to study the intraday adaptation in the context of outdoor working and non-leisure activities to understand the performance and health effects^31^.

In addition, we present the first empirical evidence that increased frequency of extremely hot temperatures induced by climate change encompasses implications not only for the quantity of activity but also for the rest-activity rhythm fundamental for environmental exposure and human metabolism. Extreme hot temperature significantly increases the proportion of daily activity taking place late at night. Delaying activity time represents both a positive adaptation (benefit) to heat stress by reducing heat exposure and a potential maladaptation (cost) in disrupting sleep patterns. Our work therefore relates to the broader discussion on the adaptation effectiveness and maladaptation^63,64^. On the one hand, such intraday adaptation can effectively reduce heat exposure at the activity time by 4–5 °C under current climate conditions. Research mapping the relationship between heat and health while neglecting this behavioral adaptation margin will overestimate the physical acclimation capacity of humans to excessive heat. On the other hand, people delay activity time by more than 25 min after 8 PM when the temperature is higher than 30 °C. Such delay can create phase delay in circadian system^65^ and thus leading to sleep disorders^33,59,66^. Future studies on the health impacts of climate change should investigate if delayed circadian rthym is a mechanism for the reduction of sleep quantity and quality on hot days.

There are several important considerations when interpreting the results of this study. First, our unit of observation is a park rather than an individual. It is possible that our findings are partially driven by sample composition changes of people going out on days with different temperatures. Future studies should track the same individuals over time and ideally incorporate individual fixed effects to ensure the results are not being biased by sample selections. Second, whether the historical relationship we built between temperature and activity will persist into the future hinges on factors in addition to temperatures, including socio-demographic compositions, built environment changes and technology evolutions. All these dynamics impose uncertainties on our projections of future climate impacts and should be explored more in future studies. Third, although our empirical analyses of historical relationships rely on hourly data, the projections of impacts from future climate change rely on daily data about climate change scenarios. The increase in daily mean temperature does not necessarily translate into an equal temperature increase at all hours within a day. This creates further uncertainties and requires researchers to develop higher-resolution climate prediction models to mitigate. Though we are unable to provide accurate future activity predictions with intraday temperature projections, we would expect future decreases in the intraday temperature range (i.e., due to a higher increase in minimum daily temperature than maximum) to further deprive the within-day adaptation margin and to create larger activity depressing effects than our current estimates. Fourth, our current analyses mainly focus on extreme temperatures. It would be a fruitful research direction for future studies to leverage data with high temporal granularity to map detailed behavioral responses to other meteorological changes and their interactions in response to future climate change. Finally, our analysis is conducted within Chinese context. It remains to be tested whether people in other countries perform identically in shifting the timing of activity in response to extreme temperatures.

To conclude, human behavioral patterns and their temporal variations are an integral part of human adaptation to our environments. Despite this, environmental and public health studies almost exclusively focus on the average level of daily activity and fail to take the timing of such activity into account. Considering within-day activity redistribution may in many settings change the dose–response function between temperature and health outcomes, alter the predictions of future climate change on activity quantity, and raise the health concerns in rest-activity rhythm disruption, which policymakers should be cognizant of. Our study adds impetus for using data with higher temporal granularity to understand intraday adaptation behaviors and enrich the environmental and behavioral indicators we used to link with human health outcomes.

## Supplementary Information

Supplementary Information.

## Data Availability

The datasets generated during and/or analysed during the current study are not publicly available due to the confidential requirements of the mobile phone positioning data. For reproducing figures and results, processed data with confidential information removed is available from the corresponding authors on reasonable request.
