# Supplementary material for: Intraday adaptation to extreme temperatures in outdoor activity
Source: Sci Rep. 2023 Jan 10;13:473. doi: 10.1038/s41598-022-26928-y (PMC9832153; doi:10.1038/s41598-022-26928-y)
Supplement: Supplementary file 1 — Supplementary Information. [file 41598_2022_26928_MOESM1_ESM.pdf]

## Supplementary Tables

**Supplementary Table 1. Summary of previous research on temperature and outdoor activities.**

| Literature | Data | Extreme cold temperature | Extreme hot temperature |
|---|---|---|---|
| (Chan and Wichman 2017) | Daily bicycle trips (bikeshare usage) | < 30 °F (~ -1 °C): -75% | > 80 °F (~ 27 °C): no sig. effect |
| (Dundas and von Haefen 2020) | Daily marine recreational fishing (survey) | < 30 °F (~ -1 °C): -22% | 80-95 °F: no sig. effect<br>>95 °F (~35 °C): -2% |
| (Graff Zivin and Neidell 2014) | Daily outdoor time (survey) | < 30 °F (~ -1 °C): - 40 min | >95 °F (~35 °C): -8 min |
| (Obradovich and Fowler 2017) | Monthly physical activity (survey) | < 0 °C: -8-9% | high temperature: no sig. effect |
| (Heaney et al., 2019) | Daily hours and distance of public bike (bikeshare usage) | Max T -10-0 °C: ~ -20% km | Max T 30-35 °C: ~ -4% km |

**Supplementary Table 2. Summary statistics.**

| Statistic | N (1) | Mean (2) | Standard Deviation (3) | Min (4) | 25% pctl (5) | 75% pctl (6) | Max (7) |
|---|---|---|---|---|---|---|---|
| Park visitation | 44,425,309 | 260.258 | 591.559 | 0 | 86 | 290 | 99,925 |
| Temperature (°C) | 44,526,965 | 17.36 | 10.593 | -41.7 | 10 | 25.7 | 48.5 |
| Air Quality Index | 44,913,818 | 72.818 | 49.647 | 1 | 42 | 88 | 500 |
| Precipitation (mm) | 44,054,119 | 0.128 | 1.146 | 0 | 0 | 0 | 133.4 |
| Wind Speed (m/s) | 44,529,555 | 2.891 | 1.639 | 0 | 1.7 | 3.7 | 34.3 |
| Relative Humidity (%) | 44,523,233 | 65.086 | 22.99 | 0 | 49 | 84 | 100 |
| Atmos. water vapor (mm) | 44,521,631 | 15.292 | 9.785 | 0 | 7 | 23.1 | 53.7 |
| Atmos. pressure (hPa) | 44,519,306 | 989.558 | 48.769 | 635 | 991 | 1,015 | 1,057 |
| Cloud coverage (%) | 45,749,465 | 49.365 | 36.717 | 0 | 9 | 87 | 100 |

Note: All variables except cloud coverage are summarized on hourly basis. An observation is a park-hour. Observations between Earlier than 6 AM or later than 11 PM are excluded.

**Supplementary Table 3. Effect of temperature on park visitation on hourly and daily resolutions.**

| | Hourly | | Daily | | |
|---|---|---|---|---|---|
| | | | Minimum | Average | Maximum |
| Temperature (°C) | (1) | Temperature (°C) | (2) | (3) | (4) |
| (-10,-5] | -0.2451*** | (-10,-5] | -0.0848*** | -0.2602*** | -0.1978*** |
| | (0.0237) | | (0.0198) | (0.0371) | (0.0294) |
| (-5,0] | -0.2105*** | (-5,0] | -0.0295 | -0.2390*** | -0.1771*** |
| | (0.0169) | | (0.0152) | (0.0290) | (0.0198) |
| (0,5] | -0.1776*** | (0,5] | 0.0193 | -0.1868*** | -0.1689*** |
| | (0.0111) | | (0.0169) | (0.0168) | (0.0175) |
| (5,10] | -0.1279*** | (5,10] | 0.0443*** | -0.1197*** | -0.127*** |
| | (0.0079) | | (0.0121) | (0.0144) | (0.0124) |
| (10,15] | -0.0770*** | (10,15] | 0.0228*** | -0.0481*** | -0.0589*** |
| | (0.0059) | | (0.0063) | (0.0088) | (0.0064) |
| (15,20] | -0.0334*** | (20,25] | -0.0679*** | 0.0466*** | 0.0565*** |
| | (0.0041) | | (0.0081) | (0.0088) | (0.0083) |
| (25,30] | 0.0039 | (25,30] | -0.1633*** | 0.0743*** | 0.1264*** |
| | (0.0034) | | (0.0179) | (0.0089) | (0.0135) |
| (30,35] | -0.0548*** | (30,35] | -0.3065*** | 0.1091*** | 0.1871*** |
| | (0.0101) | | (0.0371) | (0.0135) | (0.0112) |
| (35,40] | -0.1379*** | | | | |
| | (0.0171) | | | | |
| Hour FE | Yes | | No | No | No |
| DOW FE | Yes | | Yes | Yes | Yes |
| Park FE | Yes | | Yes | Yes | Yes |
| Month-City FE | Yes | | Yes | Yes | Yes |
| Weather Controls | Yes | | Yes | Yes | Yes |
| Observations | 43,067,963 | | 2,623,934 | 2,653,213 | 2,546,273 |
| Adjusted $R^2$ | 0.8358 | | 0.8128 | 0.8124 | 0.8125 |

Note: *p<0.01; **p<0.005; ***p<0.001. Dependent variable is ln(Park visitation +1). Control variables include air quality, precipitation, wind speed, relative humidity, atmospheric water vapor, atmospheric pressure, cloud coverage. Hourly regression has 20-25 °C as reference while daily temperature has 15-20 °C as reference. Standard errors clustered within city. In Fig.1, the regression coefficients are adjusted into percentage change by $e^{\beta} - 1$.

**Supplementary Table 4. Effects of non-temperature variables.**

| | Main Regression | | Interactions |
|---|---|---|---|
| | (1) | | (2) |
| Precipitation (mm) | -0.0073*** | (30,35] ℃ × Precipitation | 0.0003 |
| | (0.0005) | | (0.0018) |
| Wind Speed (m/s) | -0.0061*** | (35,40] ℃ × Precipitation | 0.0020 |
| | (0.0006) | | (0.0031) |
| Relative Humidity (%) | -0.0013*** | (30,35] ℃ × Wind Speed | -0.0019 |
| | (0.0002) | | (0.0020) |
| Atmos. water vapor (mm) | 0.0019* | (35,40] ℃ × Wind Speed | -0.0027 |
| | (0.0007) | | (0.0037) |
| Air Pressure (hPa) | 0.0006** | (30,35] ℃ × Humidity | 0.0006 |
| | (0.0002) | | (0.0003) |
| Cloud Coverage (%) | -0.0001 | (35,40] ℃ × Humidity | -0.0008 |
| | (0.00003) | | (0.0005) |
| Air Quality Index | 0.0001 | | |
| | (0.00004) | | |
| Temperature Bins | Yes | | Yes |
| Hour FE | Yes | | Yes |
| DOW FE | Yes | | Yes |
| Park FE | Yes | | Yes |
| Month-City FE | Yes | | Yes |
| Observations | 43,067,963 | | 43,067,963 |
| Adjusted $R^2$ | 0.8358 | | 0.8361 |

Note: *p<0.01; **p<0.005; ***p<0.001. Dependent variable is ln(Park visitation +1). Column 1 shows the regression coefficients for weather controls, with temperature bins and fixed effects included in the regression. Column 2 shows results when further adding the interaction between temperature bins and weather variables (Only interaction results for temperature bin (30,35] ℃ and (35,40] ℃ are displayed in the table, yet all the temperature bins are included for the interaction terms). Standard errors clustered within city.

**Supplementary Table 5. Heterogeneous effect of extreme temperature on park visitation.**

| Group | Sub-sample | < 0 °C<br>(1) | >30 °C<br>(2) | Observations<br>(3) | Subgroup Z-tests<br>(4) |
|---|---|---|---|---|---|
| All | all | -0.2233*** | -0.0595*** | 43,067,963 | |
| | | (0.0157) | (0.0096) | | |
| By park types | city parks/ plazas | -0.2129*** | -0.0858*** | 21,469,912 | < 0 °C: |
| | | (0.0112) | (0.0094) | | P=0.6677 |
| | tourism attractions | -0.2240*** | -0.0291** | 19,990,861 | >30 °C: |
| | | (0.0233) | (0.0091) | | P=0*** |
| By time | weekdays | -0.1752*** | -0.0517*** | 28,650,887 | < 0 °C: |
| | | (0.0096) | (0.0062) | | P=0*** |
| | weekends/ holidays | -0.2780*** | -0.0880*** | 11,294,291 | >30 °C: |
| | | (0.0179) | (0.0088) | | P=0.0007*** |
| By region | north | -0.2759*** | -0.0326 | 16,213,628 | < 0 °C: |
| | | (0.0190) | (0.0145) | | P=0.0010*** |
| | south | -0.1947*** | -0.0805*** | 26,645,175 | >30 °C: |
| | | (0.0156) | (0.0073) | | P=0.0032*** |
| By average temperature | higher than median | -0.1967*** | -0.0816*** | 27,223,458 | < 0 °C: |
| | | (0.0177) | (0.0071) | | P=0.0057*** |
| | lower than median | -0.2709*** | -0.0287 | 15,844,514 | >30 °C: |
| | | (0.0202) | (0.0138) | | P=0.0007*** |
| By park location | Rural | -0.2488*** | -0.0222 | 14,548,542 | < 0 °C: |
| | | (0.0242) | (0.0094) | | P=0.0625* |
| | Urban | -0.1992*** | -0.0765*** | 28,519,430 | >30 °C: |
| | | (0.0111) | (0.0098) | | P=0*** |
| By income | higher than median | -0.2189*** | -0.0584*** | 34,144,936 | < 0 °C: |
| | | (0.0186) | (0.0113) | | P=0.6744 |
| | lower than median | -0.2087*** | -0.0794*** | 7,540,220 | >30 °C: |
| | | (0.0156) | (0.0069) | | P=0.1127 |

Note: *p<0.01; **p<0.005; ***p<0.001. Dependent variable is ln(Park visitation +1). Control variables include air quality, precipitation, wind speed, relative humidity, atmospheric water vapor, atmospheric pressure, cloud coverage. Hour, day-of-week, city-by-month and park fixed effects are included in the regressions. 20-25 °C bin is set as reference. Standard errors clustered within city. In Fig.2, the regression coefficients are adjusted into percentage change by $e^{\beta} - 1$. Column 4 shows the z-test (reference) results across groups within each category to test whether there are statistically significant differences between coefficients.

**Supplementary Table 6. Daily average temperature and activity in different times of the day.**

| Daily Average Temperature (℃) | All times (1) | Morning/ Night (2) | Noon/ Evening (3) |
|---|---|---|---|
| (-10,-5] | -0.1934*** | -0.1844*** | -0.1999*** |
| | (0.0290) | (0.0272) | (0.0309) |
| (-5,0] | -0.1676*** | -0.1657*** | -0.1692*** |
| | (0.0238) | (0.0238) | (0.0242) |
| (0,5] | -0.1166*** | -0.1184*** | -0.1172*** |
| | (0.0091) | (0.0100) | (0.0087) |
| (5,10] | -0.0669*** | -0.0684*** | -0.0677*** |
| | (0.0050) | (0.0059) | (0.0048) |
| (10,15] | -0.0286*** | -0.0333*** | -0.0262*** |
| | (0.0038) | (0.0031) | (0.0045) |
| (20,25] | 0.0336*** | 0.0526*** | 0.0198*** |
| | (0.0053) | (0.0056) | (0.0052) |
| (25,30] | 0.0324*** | 0.0796*** | -0.0026 |
| | (0.0069) | (0.0071) | (0.0075) |
| (30,35] | 0.0128 | 0.0927*** | -0.0468*** |
| | (0.0102) | (0.0098) | (0.0118) |
| (35,40] | -0.0488*** | 0.0511*** | -0.1225*** |
| | (0.0135) | (0.0124) | (0.0166) |
| Hour FE | Yes | Yes | Yes |
| DOW FE | Yes | Yes | Yes |
| Park FE | Yes | Yes | Yes |
| Month-City FE | Yes | Yes | Yes |
| Observations | 43,147,335 | 20,324,544 | 22,822,791 |
| Adjusted $R^2$ | 0.8348 | 0.8389 | 0.8652 |

Note: *p<0.01; **p<0.005; ***p<0.001. Dependent variable is ln(Park visitation +1). Sub-samples are restricted by time periods and regressions are run separately for each sub-sample. Control variables include air quality, precipitation, wind speed, relative humidity, atmospheric water vapor, atmospheric pressure, cloud coverage. 15-20 ℃ bin is set as reference. Standard errors clustered within city. In Fig.3a, the regression coefficients are adjusted into percentage change by $e^{\beta} - 1$.

**Supplementary Table 7. Within-day hourly activity distribution patterns.**

| Time | >30 ℃ (1) | 25-30 ℃ (2) | 20-25 ℃ (3) | 15-20 ℃ (4) |
|---|---|---|---|---|
| 6 AM | -0.6277*** | -0.6542*** | -0.6951*** | -0.7900*** |
| | (0.0154) | (0.0185) | (0.0210) | (0.0238) |
| 7 AM | -0.2471*** | -0.2699*** | -0.2860*** | -0.3317*** |
| | (0.0134) | (0.0150) | (0.0137) | (0.0138) |
| 8 AM | -0.0430*** | -0.0692*** | -0.0862*** | -0.1219*** |
| | (0.0071) | (0.0088) | (0.0088) | (0.0086) |
| 9 AM | 0.0360*** | 0.013 | -0.0062 | -0.0371*** |
| | (0.0055) | (0.0053) | (0.0061) | (0.0059) |
| 10 AM | 0.0762*** | 0.0644*** | 0.0477*** | 0.0249*** |
| | (0.0072) | (0.0051) | (0.0055) | (0.0050) |
| 11 AM | 0.0774*** | 0.0684*** | 0.0555*** | 0.0413*** |
| | (0.0061) | (0.0045) | (0.0046) | (0.0038) |
| 12 PM | 0.0420*** | 0.0360*** | 0.0279*** | 0.0208*** |
| | (0.0034) | (0.0024) | (0.0028) | (0.0023) |
| 2 PM | 0.0175*** | 0.0220*** | 0.0226*** | 0.0277*** |
| | (0.0029) | (0.0025) | (0.0023) | (0.0028) |
| 3 PM | 0.0387*** | 0.0417*** | 0.0389*** | 0.0432*** |
| | (0.0047) | (0.0043) | (0.0035) | (0.0041) |
| 4 PM | 0.0746*** | 0.0696*** | 0.0559*** | 0.0496*** |
| | (0.0061) | (0.0059) | (0.0054) | (0.0059) |
| 5 PM | 0.1127*** | 0.0850*** | 0.0495*** | 0.0231* |
| | (0.0083) | (0.0091) | (0.0082) | (0.0084) |
| 6 PM | 0.1208*** | 0.0623*** | 0.0003 | -0.0476*** |
| | (0.0118) | (0.0131) | (0.0107) | (0.0113) |
| 7 PM | 0.1172*** | 0.0342 | -0.0440*** | -0.1049*** |
| | (0.0164) | (0.0158) | (0.0122) | (0.0125) |
| 8 PM | 0.0818*** | -0.0048 | -0.0883*** | -0.1536*** |
| | (0.0240) | (0.0216) | (0.0166) | (0.0167) |
| 9 PM | -0.0443 | -0.1230*** | -0.2035*** | -0.2603*** |
| | (0.0275) | (0.0249) | (0.0190) | (0.0196) |
| 10 PM | -0.2953*** | -0.3598*** | -0.4283*** | -0.4698*** |
| | (0.0280) | (0.0275) | (0.0222) | (0.0228) |
| Hour FE | No | No | No | No |
| DOW FE | Yes | Yes | Yes | Yes |
| Park FE | Yes | Yes | Yes | Yes |
| Month-City FE | Yes | Yes | Yes | Yes |
| Observations | 3,744,994 | 8,320,998 | 8,017,164 | 6,826,441 |
| Adjusted $R^2$ | 0.8809 | 0.8693 | 0.8599 | 0.8499 |

Note: *p<0.01; **p<0.005; ***p<0.001. Dependent variable is ln (Park visitation +1). Subsamples are based on average daily temperature and regressions are run for each sample separately. Control variables include air quality, precipitation, wind speed, relative humidity, atmospheric water vapor, atmospheric pressure, cloud coverage. 1 PM is set as the reference. Each time point represents the following hour (e.g., 6 AM represents 6 AM to 7 AM). Standard errors clustered within city. In Fig.4a, the regression coefficients are adjusted into percentage change by $e^{\beta} - 1$.

## Supplementary Figures

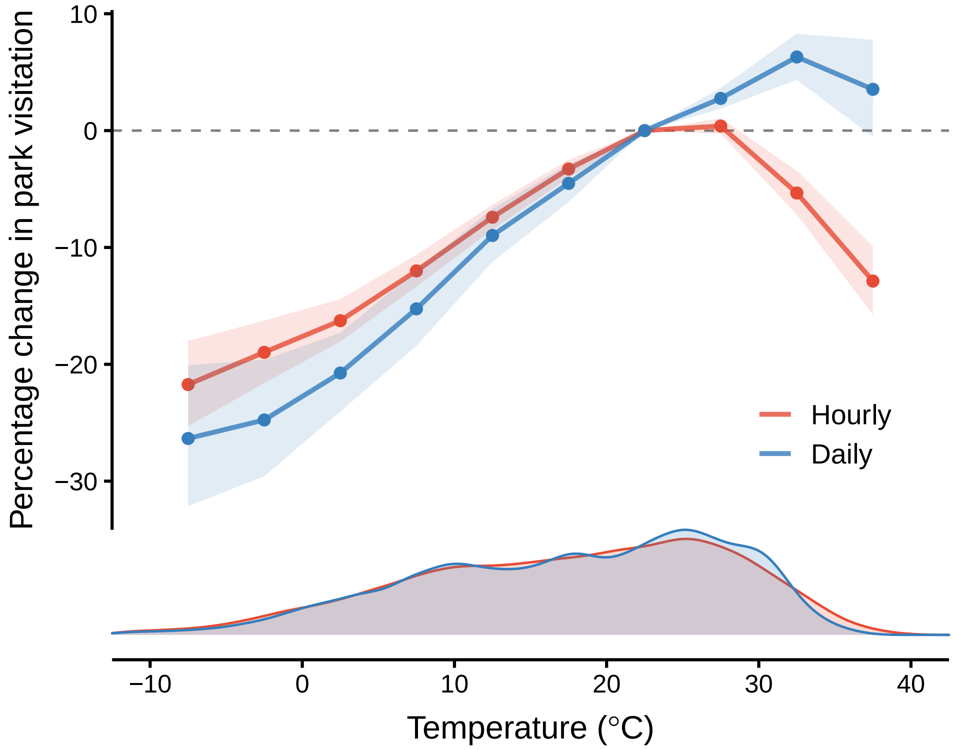


**Supplementary Figure 1. Effects of daily temperature from different time granularity**. Estimation outputs from daily and hourly observations are presented respectively. Shaded areas are 95% confidence intervals. The density plots at the bottom of each figure show the share of parks exposed to different temperatures in the sample used to estimate each curve.

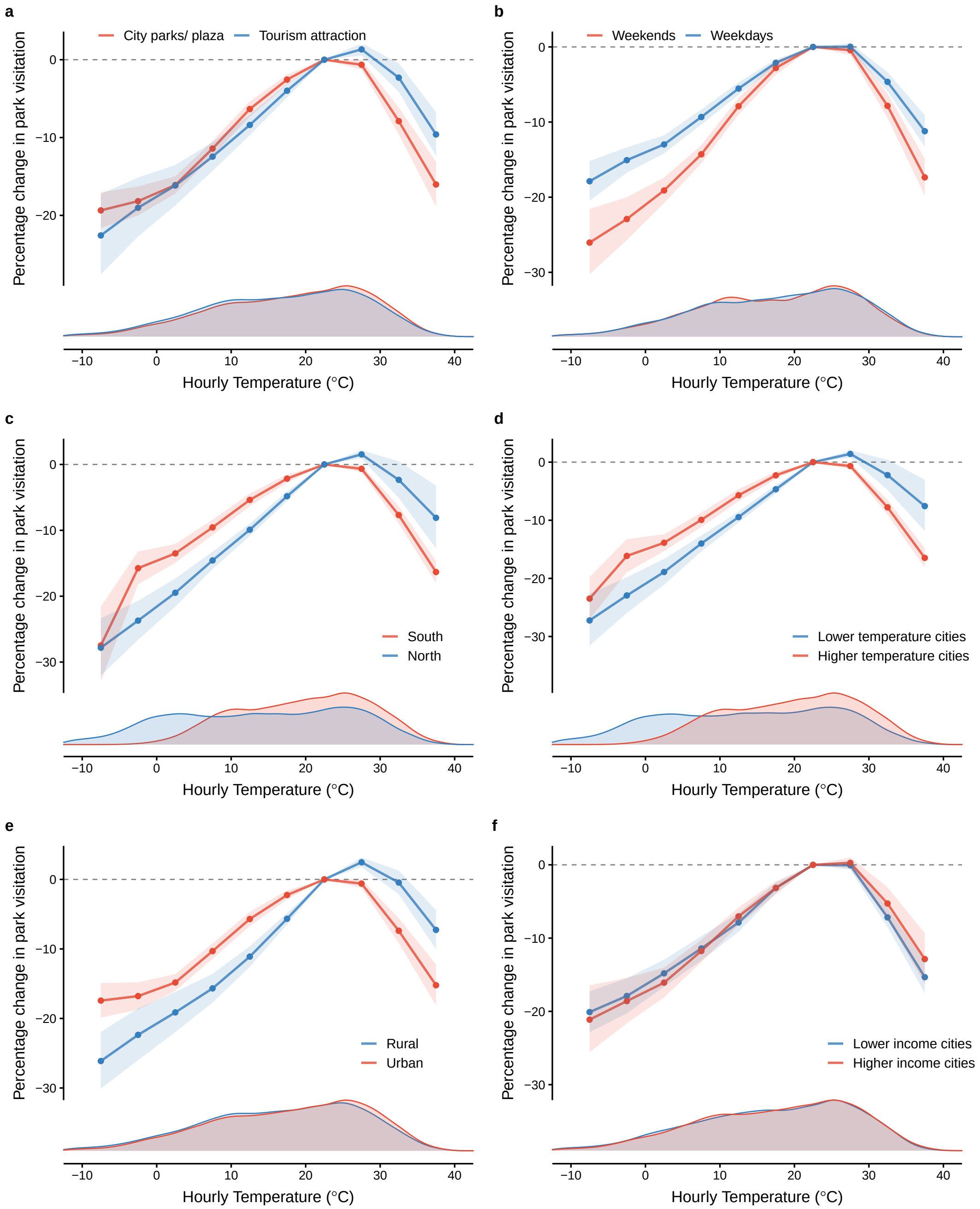


**Supplementary Figure 2. Heterogeneity results for each temperature bin**. **a**, by park types. **b**, by time. **c**, by geographical regions. **d**, by city custom temperature level. **e**, by park location (urban/ rural). **f**, by city income. Shaded areas are 95% confidence intervals. The density plots at the bottom of each figure show the share of the park exposed to different temperatures in the sample used to estimate each curve.

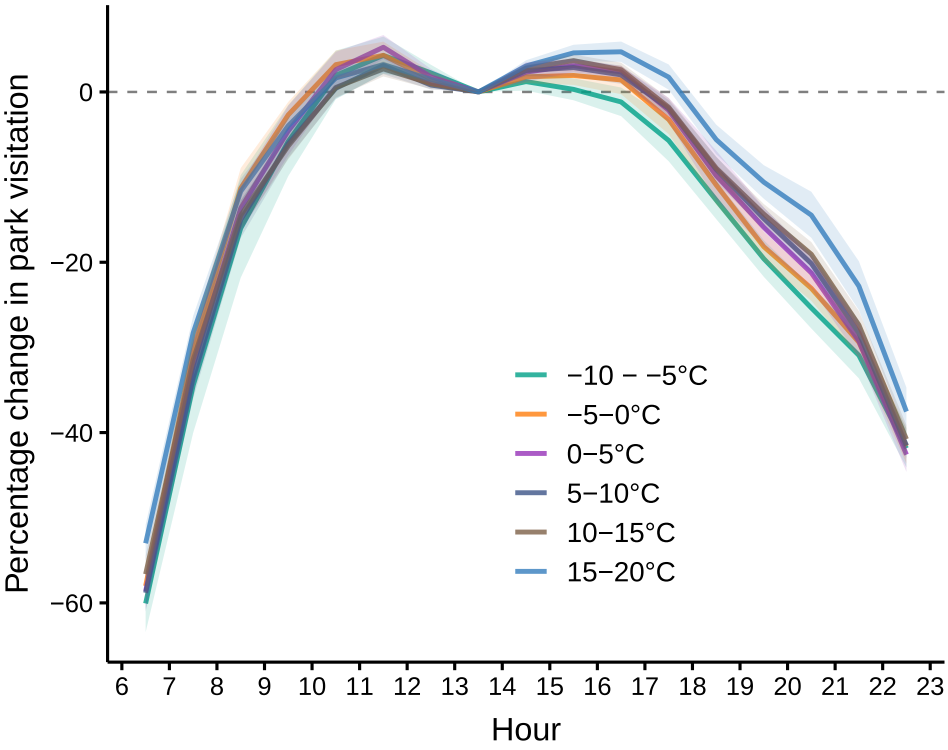


**Supplementary Figure 3. Effects of cold temperature on the within-day activity distribution pattern**. Each line represents the within-day activity pattern for days lying in each daytime average temperature range. Shaded areas are 95% confidence intervals.

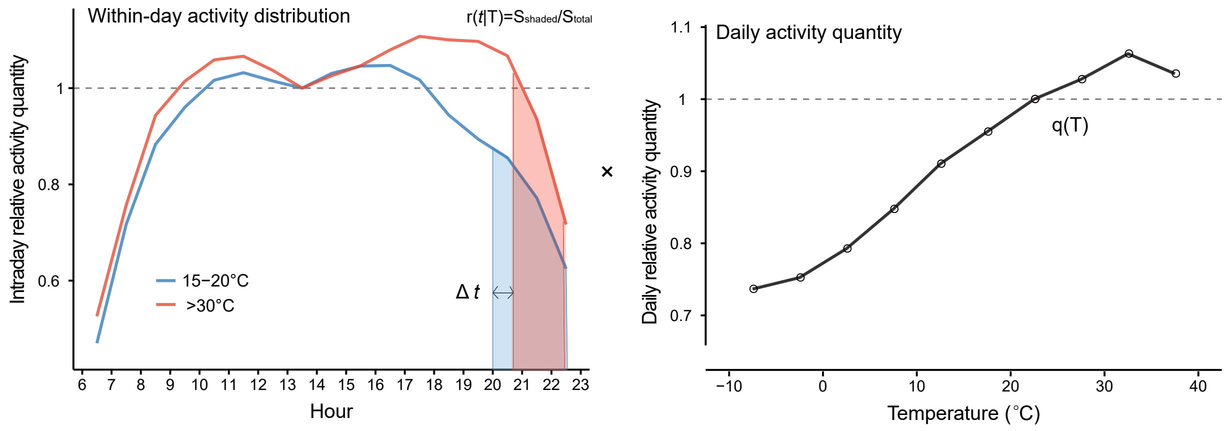


**Supplementary Figure 4. Schematic illustration of the definition of time shift and its calculation procedure**. Within-day distribution daily quantity results are from the regression results of Fig. 4a and Supplementary Figure 1. Equalizing activity share times activity quantity under different temperatures produces the time shift function in Fig. 4b.

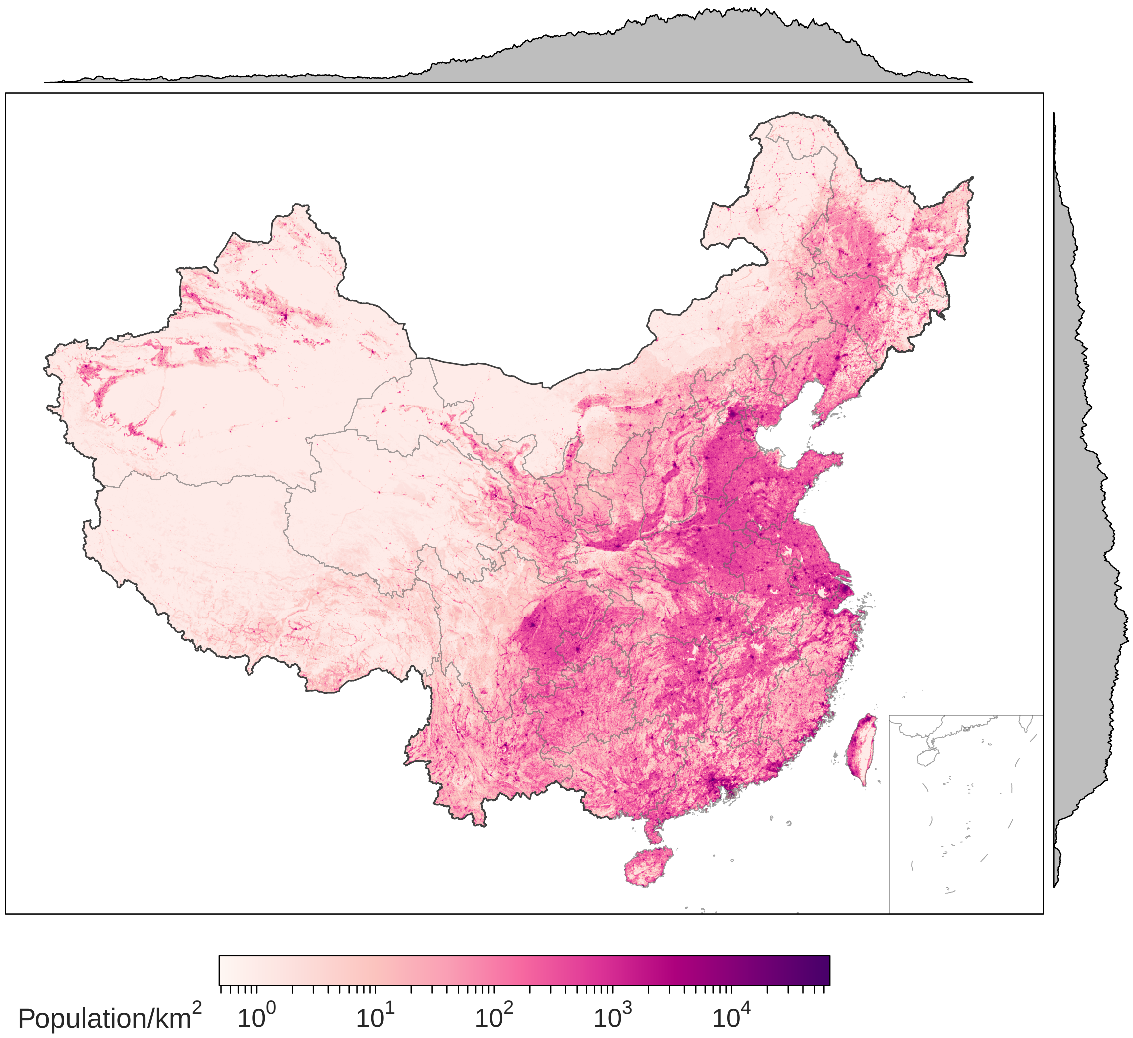


**Supplementary Figure 5. Distribution of population density of China in 2017**. The Qinghai-Tibet Plateau in west China is the only region where climate change has positive human activity impacts, yet with a very limited population. The grid population data is from LandScan: https://landscan.ornl.gov/.

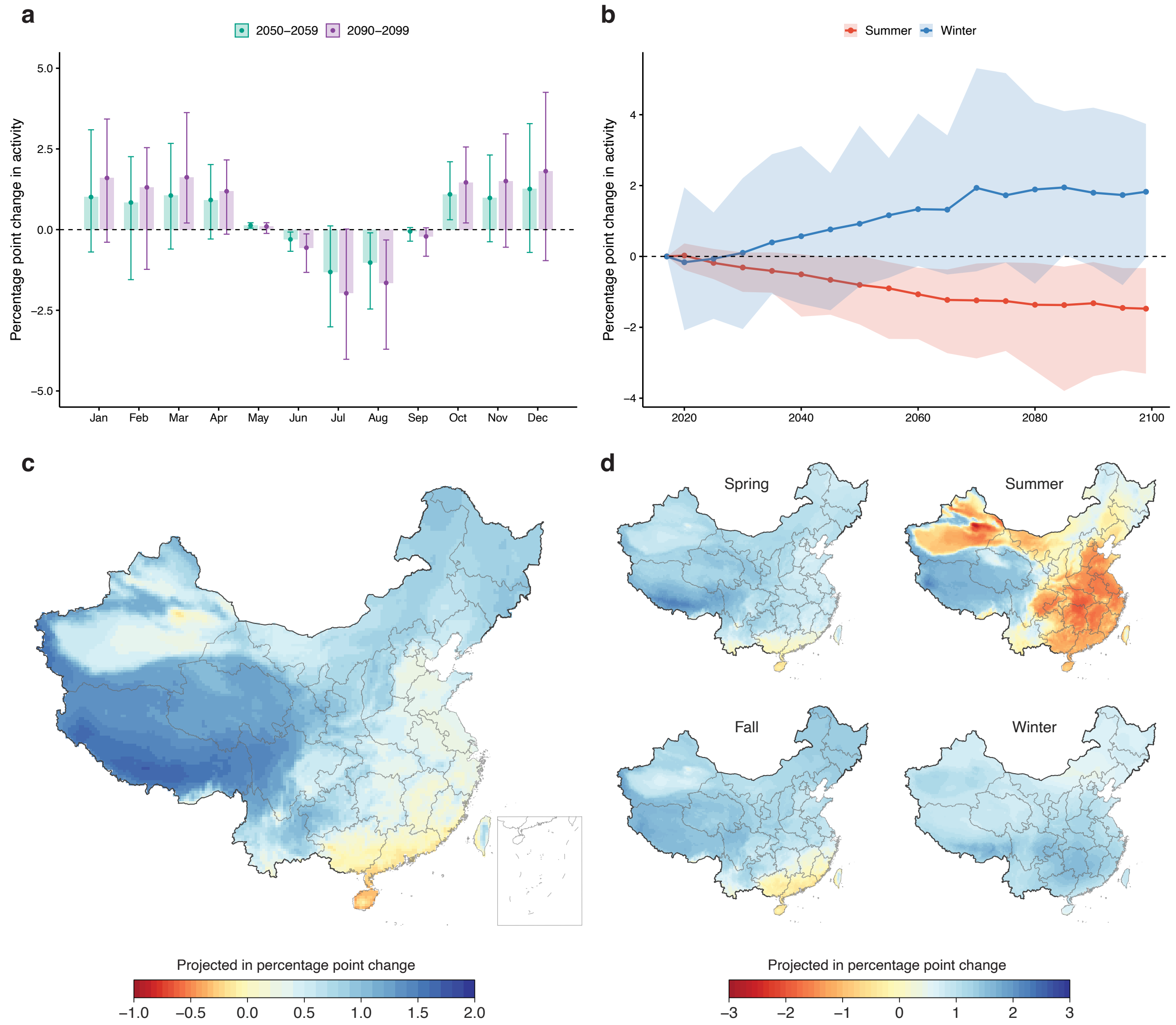


**Supplementary Figure 6. Projected change in park visitation quantity under RCP4.5 scenario**. **a**, Additional percentage point change in park visitation quantity by month in the year 2050 and 2099 compared with 2017 due to climate change. **b**, The percentage change in activity quantity for summer and winter respective for all years in the future. Geographic estimates of the projected change in park visitation quantity in all areas of China in 2090-2099. Error bars and shaded areas represent 95% confidence intervals calculated through Monte Carlo sampling of econometric parameter estimates and climate models. **c**, Geographic dispersion of the predicted additional yearly average effects of climate change-induced activity change in 2090-2099. **d**, Prediction of additional climate change-induced activity change in 2090-2099 by seasons.

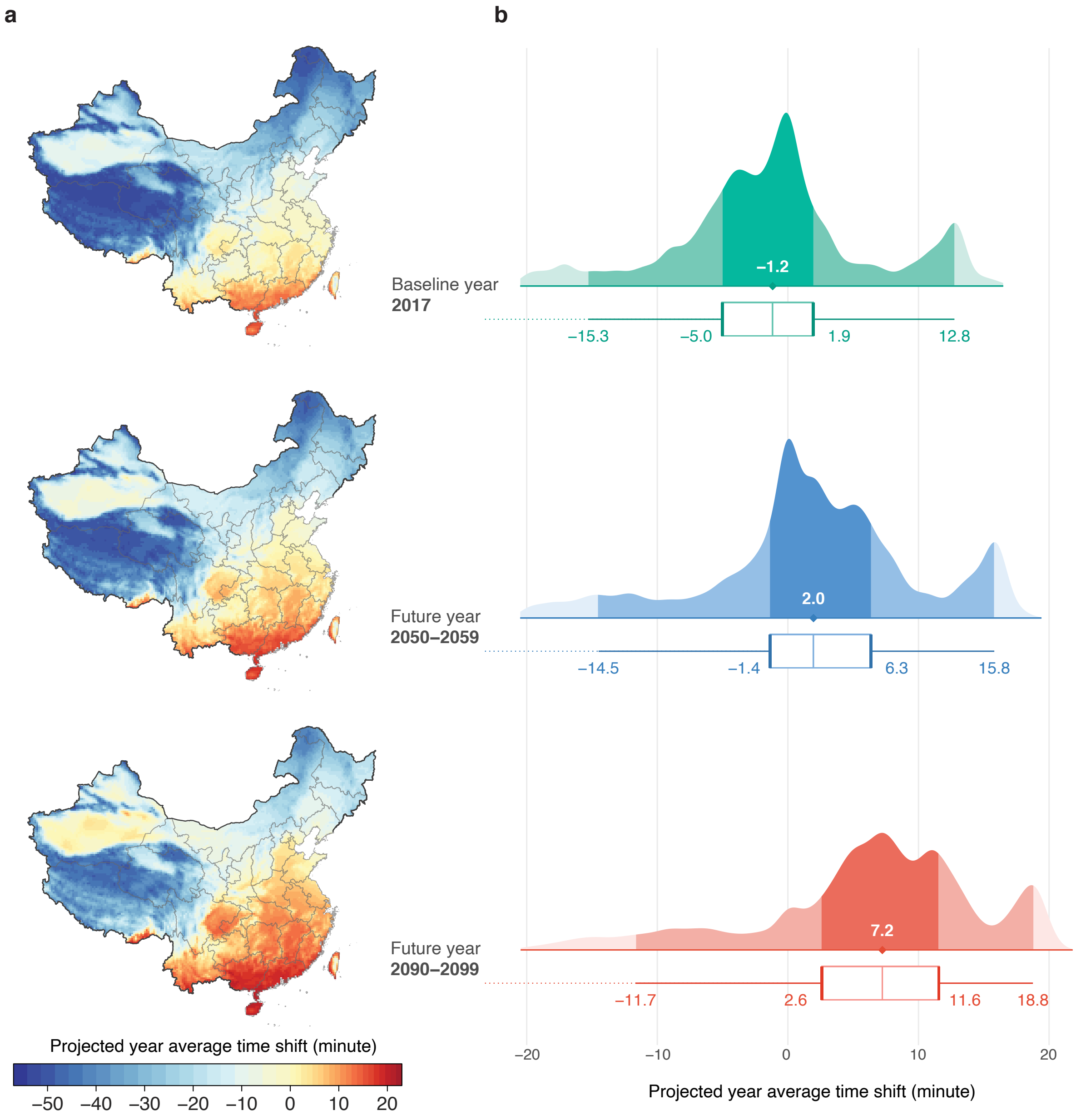


**Supplementary Figure 7. Projected time shift in intraday timing of activity under RCP8.5.** **a**, Distribution of average yearly activity time shift due to temperature in summer of 2017, at mid-century (2050-2059) and end of the century (2090-2099) under RCP8.5 scenario. **b**, Densityplot of the size of populations subjecting to each yearly average time shift levels.

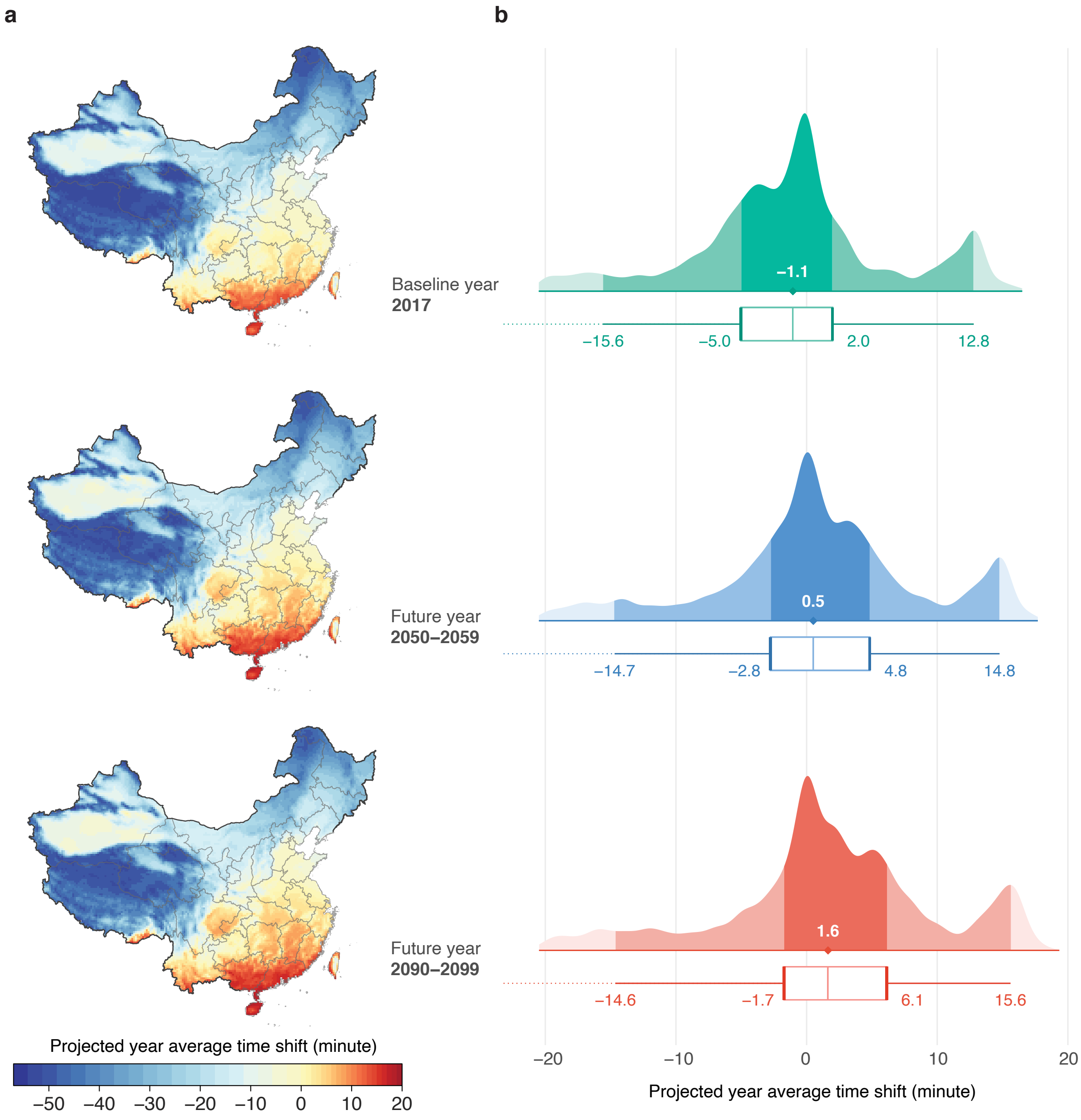


**Supplementary Figure 8. Projected time shift in intraday timing of activity under RCP4.5.** **a**, Distribution of average yearly activity time shift due to temperature in 2017, at mid-century (2050-2059) and end of the century (2090-2099) under RCP4.5 scenario. **b**, Densityplot of the size of populations subjecting to each yearly average time shift levels.

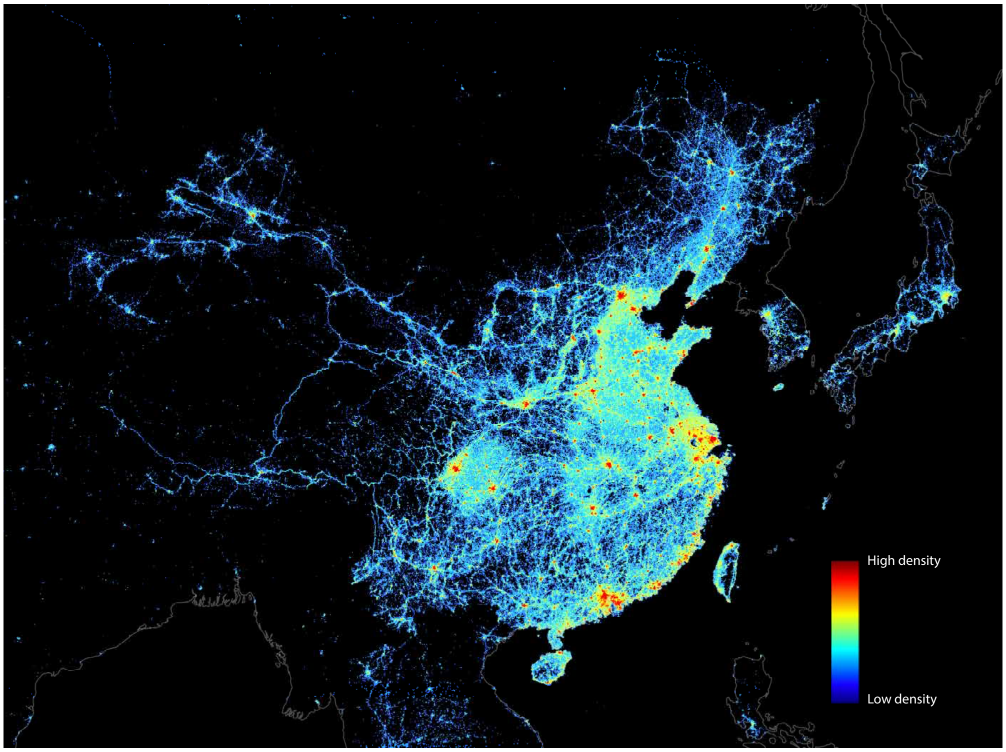


**Supplementary Figure 9. Main datasets**. Cell-phone location data heatmap.

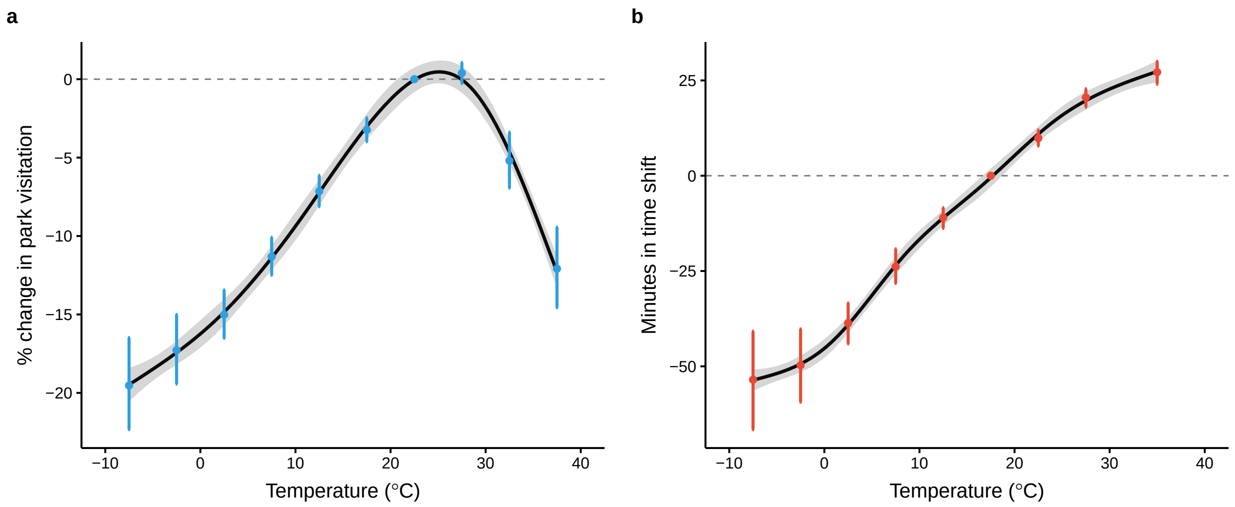


**Supplementary Figure 10. Natural cubic spline fitted models**. **a**, Hourly temperature and percentage change in activity quantity; **b**, Daily average temperature and activity time shift. Dots and error bars show point estimates and 95% confidence intervals for each temperature bin. The dark lines connecting the dots are smoothed through the Loess algorithm.
